# Supplementary material for: Determinants of socioeconomic factors for quality of life and depressive symptoms in community-dwelling older people: A cross-sectional study in Brazil and Portugal
Source: PLoS One. 2023 Jun 13;18(6):e0287163. doi: 10.1371/journal.pone.0287163 (PMC10263313; doi:10.1371/journal.pone.0287163)
Supplement: Attachment — Submitted filename: Response to Reviewers.docx [file pone.0287163.s001.docx]

**Response to Reviewers**

**Reviewer #1:**

The manuscript presents an important theme that contributes to the study of geriatric depression in relation to socioeconomic factors. The authors present an adequate literature review that discusses in depth all the most relevant elements. I have only small suggestions that can be changed to improve understanding of what has been presented.

General

- Throughout the text, the abbreviation for Quality of Life should be changed to “QoL” instead of “QOL”.

**Answer: Corrections regarding abbreviations have been made.**

Results

- In tables 1, 2, 3, 4 and 5, some data is bold. The reason for this is not very clear. I suggest that authors enter this information in the legend of each table or delete this highlight.

**Answer: Regarding the bold in some values, we would like to visually highlight the results with statistical significance. However, in view of your note, we prefer to remove it to avoid confusion for readers. Thanks.**

**Reviewer #2:**

Dear Author,

Congratulations on this well-written manuscript. My comments are as follows:

1. Line 45 (Suggestion): Change "/" from "(76.0%/p = 0.224)" to "(76.0%, p = 0.224)"

**Answer: Change made.**

2. Line 86, 91 and 94..: Check grammar throughout manuscript. Position of full stop should be consistent with citation - etc., change "(QOL)[4]." to "(QOL).[4]"

**Answer: The model sent by the Plos One journal advises that the period should be after the citation. However, there must be a space between the last word and the quote. We have made this change and believe this resolves this issue.**

3. Introduction: It would be helpful for the readers if the authors were to define active ageing and how it relates to the overall conceptual framework of this study

**Answer: We added a paragraph about that. Thanks for the suggestion.**

4. Line 298-300...: There are a few capitalised words which I think would be better left lower case; similar to other words in the discussion - please check

**Answer: We checked and made the necessary corrections.**

5. Line 314-317 would fit better narratively in the introduction

**Answer: The concepts brought in lines 314-317 refer to specific variables of the SF-36 and we thought it best to keep this text in the discussion. However, we thought it worth adding this relationship in the introduction and we did.**

6. Line 327: Double check the word "elderly people", it would be more appropriate to use "older adults/people" Some examples of biased language are “elder” or “elderly”, “senior”, or “the aged”. It is advisable against using these terms because they evoke negative stereotypes of older adults, which can lead to othering older adults, bias against older adults, and poor outcomes for older adults.

**Answer: We standardized the use of the term “older people” throughout the text.**

Thank you.
